# Adenine Dinucleotide Second Messengers and T-lymphocyte Calcium Signaling

**DOI:** 10.3389/fimmu.2013.00259

**Published:** 2013-08-29

**Authors:** Insa M. A. Ernst, Ralf Fliegert, Andreas H. Guse

**Affiliations:** ^1^The Calcium Signalling Group, Department of Biochemistry and Molecular Cell Biology, University Medical Center Hamburg-Eppendorf, Hamburg, Germany

**Keywords:** calcium signaling, T-lymphocyte, calcium release, nicotinic acid adenine dinucleotide phosphate, cyclic ADP-ribose, adenosine diphosphoribose, TRPM2 cation channels, calcium entry

## Abstract

Calcium signaling is a universal signal transduction mechanism in animal and plant cells. In mammalian T-lymphocytes calcium signaling is essential for activation and re-activation and thus important for a functional immune response. Since many years it has been known that both calcium release from intracellular stores and calcium entry via plasma membrane calcium channels are involved in shaping spatio-temporal calcium signals. Second messengers derived from the adenine dinucleotides NAD and NADP have been implicated in T cell calcium signaling. Nicotinic acid adenine dinucleotide phosphate (NAADP) acts as a very early second messenger upon T cell receptor/CD3 engagement, while cyclic ADP-ribose (cADPR) is mainly involved in sustained partial depletion of the endoplasmic reticulum by stimulating calcium release via ryanodine receptors. Finally, adenosine diphosphoribose (ADPR) a breakdown product of both NAD and cADPR activates a plasma membrane cation channel termed TRPM2 thereby facilitating calcium (and sodium) entry into T cells. Receptor-mediated formation, metabolism, and mode of action of these novel second messengers in T-lymphocytes will be reviewed.

Adenine derived Ca^2+^ mobilizing second messengers comprise nicotinic acid adenine dinucleotide phosphate (NAADP), cyclic ADP-ribose (cADPR), and adenosine diphosphoribose (ADPR; Figure 1). They are all metabolites of nicotinamide adenine dinucleotide (NAD), a dinucleotide well known as coenzyme of oxidoreductases. NAD is converted by the multifunctional ectoenzyme NAD-glycohydrolase/ADP-ribosyl cyclase CD38 to ADPR and cADPR (Figure 1). The fact that the active site of CD38 faces the extracellular space while the targets for its products are located inside the cell, also known as topological paradox (1), has recently been investigated in detail. Importantly, in addition to the type II conformation with the active site facing the extracellular space, it was demonstrated that a smaller portion of CD38 is expressed in type III conformation thereby allowing for production of ADPR and cADPR within the cytosol (2). Another interesting feature of CD38 is the fact that it not only can make cADPR and ADPR, but also can synthesize NAADP, at least *in vitro* (Figure 1). However, this base-exchange mechanism needs nicotinamide adenine dinucleotide phosphate (NADP) and an excess of nicotinic acid as substrates and it works at acidic pH. Thus, it remains unclear whether this reaction is of physiological significance for second messenger formation in the cytosol. The substrate for the base-exchange reaction, NADP, is produced from NAD by NAD kinase (3). While mature, naïve T cells express only small amounts of CD38, it is upregulated as a consequence of mitogenic stimulation (4, 5). This is for instance seen after infection with HIV in activated antiviral CD8^+^ T cells (6). CD38 expression in the CD8 compartment is therefore used to monitor antiretroviral therapy (7). Whether CD38 upregulation in activated T cells affects Ca^2+^ signaling compared to naïve, mature T cells is not known, but it is easy to envision the production of Ca^2+^ mobilizing messengers in effector cells being facilitated by upregulation of CD38, allowing for faster Ca^2+^ responses necessary for secretion of cytokines or granzymes and perforin in contrast to activation of calcineurin and NFAT in naïve cells.

**Figure 1:**
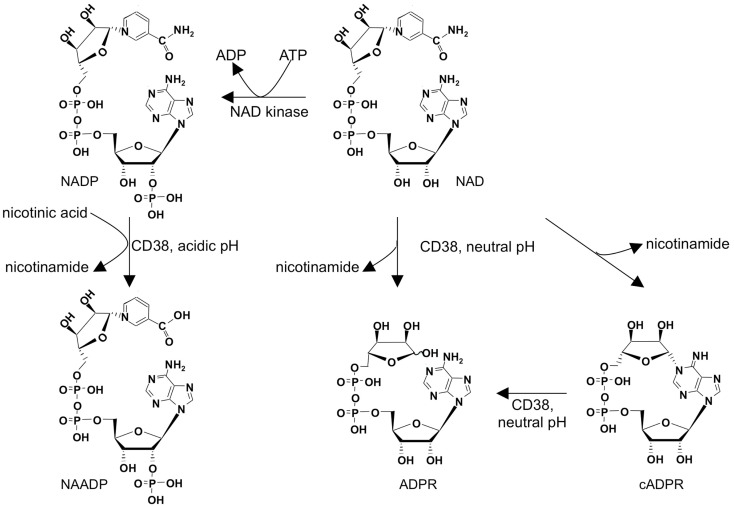
**Metabolism of adenine derived second messengers cADPR, NAADP, and ADPR**.

Ca^2+^ signaling is one of the essential intracellular signaling pathways involved in T cell activation. It has long been known that both Ca^2+^ release and Ca^2+^ entry contribute to global Ca^2+^ signaling in T cells. In addition to Ca^2+^ release and Ca^2+^ entry evoked by the adenine derived Ca^2+^ mobilizing second messengers introduced above, two “standard” Ca^2+^ signaling systems are involved: (i) Ca^2+^ release by D-*myo*-inositol 1,4,5-trisphosphate [IP_3_; (8)] and store-operated or capacitative Ca^2+^ entry (9). Since these systems have been thoroughly investigated and described in detail, they will not be reviewed in this article. However, due to their importance for T cell Ca^2+^ signaling, their roles will be mentioned and/or depicted, as for example in Figure 2.

**Figure 2:**
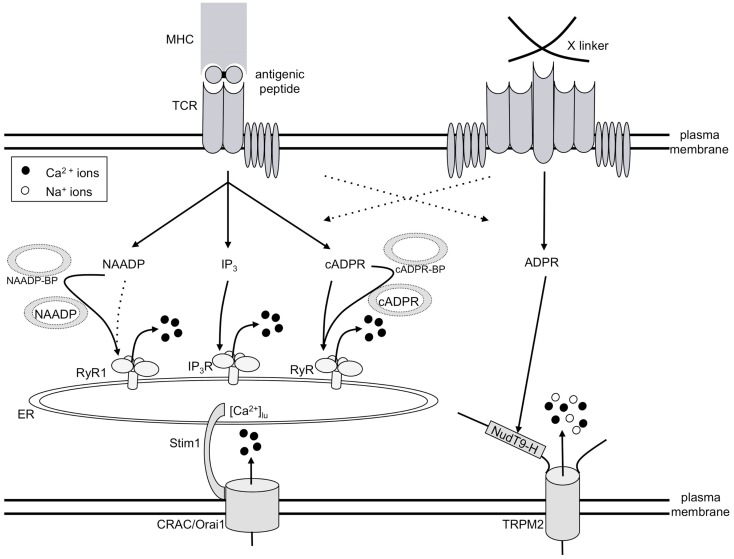
**Model of T cell Ca^2+^ signaling**. TCR/CD3 ligation by antigenic peptide presented by MHC molecules on antigen presenting cells results in consecutive formation of the second messengers NAADP, IP_3_, and cADPR, all of which release Ca^2+^ from the ER. Thus, a continuously decreased intraluminal free Ca^2+^ concentration in the ER ([Ca^2+^]_lu_) resulting from this constant Ca^2+^ release concomitantly activates CRAC/Orai1 channels in the plasma membrane. The mode of action of both NAADP and cADPR likely involves specific binding proteins for both second messengers (abbreviated here as NAADP-BP or cADPR-BP). A strong stimulus, e.g., cross-linking of receptors by concanavalin A (right side of Figure 2), triggers formation of ADPR and activation of TRPM2, in addition to the mechanisms described on the left side of the figure.

The initial player in our model of T cell Ca^2+^ signaling is NAADP (Figure 2) being formed within seconds upon TCR/CD3 ligation (10). However, NAADP is a rather short-lived second messenger, although after a rapid decrease to control levels, a second much smaller rise over several minutes was observed in Jurkat T cells (11). NAADP probably delivers the first local Ca^2+^ signals which then act as co-agonists at IP_3_ receptors (IP_3_R) and ryanodine receptors (RyR). IP_3_ is formed soon after the initial NAADP peak (12) and releases Ca^2+^ via IP_3_R (13). Finally, cADPR starts to increase and acts on RyR (14); likely, Ca^2+^ released by NAADP and/or IP_3_ facilitates the action of cADPR. Continuous Ca^2+^ release by these consecutively increased second messengers results in continuously decreased luminal Ca^2+^ concentration in the ER ([Ca^2+^]_lu_). Stromal interaction molecule-1 (Stim1) senses the decreased [Ca^2+^]_lu_ and activates Ca^2+^ entry via orai/CRAC channels (15–17).

In addition to this Ca^2+^ signaling pathway involved in T cell activation or re-activation, high input signal strength, e.g., obtained by a high concentration of the cross-linking lectin Concanavalin A, activates another different Ca^2+^ entry system operated by ADPR and the transient receptor potential channel, subtype melastatin 2 (TRPM2; Figure 2).

Following we will review hallmarks of NAADP, cADPR, and ADPR as second messengers in T cell Ca^2+^ signaling.

## NAADP

Upon activation of the TCR/CD3 complex, formation of NAADP rapidly increases within 10–20 s in Jurkat T cells. Following a subsequent decrease within the first minute, a continuously elevated [NAADP] remains for 5–20 min (10). It has been proposed that NAADP may act as an early triggering messenger, mediating initial localized Ca^2+^ events which are subsequently amplified to a global signal, e.g., by recruitment of further channels, other second messengers like cADPR, IP_3_, and/or Ca^2+^ induced Ca^2+^ release (CICR). In T-lymphocytes a bell-shaped concentration-response curve following NAADP microinjection is observed. Compared to other second messengers such as IP_3_, already low concentrations in the nanomolar range (30–100 nM) induce Ca^2+^ signaling in T-lymphocytes (18).

The mechanism and very early kinetics of receptor-mediated formation of NAADP *in vivo* remains unclear and has been discussed previously [e.g., (19)]. In brief, NAADP is formed *in vitro* by a base-exchange of NADP in presence of nicotinic acid and a pH of 5 [Figure 1; (20)]. Further, at pH 5, but also at pH 7.4, 2′-phospho-cADPR may be converted to NAADP (21). Both reactions are catalyzed by the membrane bound, multifunctional enzyme CD38 and require presence of up to millimolar concentrations of nicotinic acid *in vitro* (20, 21). Furthermore, influx of extracellular NAADP may also induce Ca^2+^ signals as shown in a rat basophilic cell line (22). Interestingly, gene silencing of CD38 in Jurkat T-lymphocytes did not result in decreased NAADP levels. Rather, in thymus and spleen of CD38 knock-out mice increased NAADP levels were observed, thus indicating that CD38 may particularly drive degradation of NAADP (23, 24). Accordingly, in T cells to date CD38 may be primarily understood as degrading enzyme while its role in NAADP synthesis *in vivo* remains to be elucidated. In T-lymphocytes and other CD38^+^ cells, NAADP is degraded to 2′-phospho-ADPR at neutral and acidic pH by CD38, but degradation may also occur non-specifically via nucleotide pyrophosphatases (25).

The targeted receptor(s) and hence target organelle(s) of NAADP are still under debate (19, 26–29). In general, RyR have been implicated as NAADP targets in different cell types, e.g., skeletal muscle cell (30), or pancreatic acinar cells (31, 32). Nonetheless, in the majority of mammalian cells as well as in sea urchin eggs there is evidence that NAADP may primarily target acidic stores and that the RyR located on the ER may rather play a central role in the amplification of the Ca^2+^ signal (33–35).

However focusing particularly on data obtained in T-lymphocytes, NAADP Ca^2+^ signaling strongly depends on RyR activity in the ER (36–40). Following either knock-down or inhibition of RyR by ryanodine in Jurkat T cells, subcellular and global Ca^2+^ signals by NAADP microinjection were inhibited or almost completely abolished (36, 38). In primary effector T-lymphocytes the NAADP antagonist BZ194 inhibits Ca^2+^ signaling, e.g., during formation of the immunological synapse. Furthermore, BZ194 has been shown to selectively inhibit NAADP dependent binding of [^3^H]ryanodine to RyR1 (39). Interestingly, in primary effector T cells of an animal model of multiple sclerosis, experimental autoimmune encephalomyelitis (EAE), BZ194 leads to a decrease in cell motility and invasive capacity as well as a decrease in cytokine expression, all of which indicate the central role of NAADP-mediated Ca^2+^ signaling in T cells possibly via RyR (40). In contrast to these results obtained in CD4^+^ T cells, in cytotoxic T cells NAADP appears to target two-pore channels (TPC) on cytolytic granules (41). In general, overexpression or inhibition of the endolysosomal TPC1 and TPC2 suggest that NAADP initiates Ca^2+^ events via TPC [e.g., (42–44)]. Recently, the N-terminus of TPC1 has been identified as functional region for NAADP-mediated Ca^2+^ signaling (26). In contrast, it was shown that NAADP-mediated Ca^2+^ signaling in TPC1^−/−^/TPC2^−/−^ mice does not differ from wild-type mice (27). Thus, whether NAADP primarily targets TPCs is controversial and particularly the effect of NAADP on TPCs in T-lymphocytes is not yet clear.

Further, in T-lymphocytes and neutrophils TRPM2 is activated by micromolar concentrations of NAADP *per se*, but particularly in synergism with cADPR (45, 46). The effect of cADPR on TRPM2 however, could not be confirmed in HEK293 cells overexpressing TRPM2 and contamination of commercial cADPR preparations with ADPR have been discussed (47, 48). NAADP has been shown to target the unspecific Ca^2+^ channel TRPML1 in smooth muscle myocytes (49). Whether TRPML1 and TRPM2 are of functional relevance within NAADP – mediated Ca^2+^ signaling in T-lymphocytes, remains to be elucidated.

Despite the questions which organelles are targeted by NAADP and which specific downstream mechanisms may underlie the initiated Ca^2+^ events, also the identity of the NAADP receptor remains unclear. Photoaffinity labeling in mammalian cells using a probe specific for NAADP binding proteins was not altered upon overexpression or knock-out of TPC1 or TPC2, but suggests that a yet not identified 22/23 kDa protein binds NAADP and may hence couple NAADP to its respective Ca^2+^ channels (50, 51), a mechanism recently introduced as unifying hypothesis of NAADP action (29).

## Cyclic ADP-Ribose

Cyclic adenosine diphosphoribose (cADPR) was the first Ca^2+^ mobilizing second messenger discovered as derivative of an adenine dinucleotide (52, 53). Though first described in sea urchin egg homogenates, the Ca^2+^ mobilizing activity of cADPR was soon detected in many cells types. In 1995 we published the first report demonstrating specific Ca^2+^ release in human Jurkat T cells (54). Central aspects of the role of cADPR in T cell Ca^2+^ signaling were subsequently published by our laboratory: (i) formation of cADPR upon TCR/CD3 ligation (14, 55), and (ii) mode of action of cADPR by activation of Ca^2+^ release via RyR, as shown by gene silencing of RyR (56). Further, we demonstrated tyrosine phosphorylation of RyR upon TCR/CD3 ligation; in permeabilized T cells enhancement of cADPR evoked Ca^2+^ release by tyrosine kinase p59fyn was observed (57). Importantly, we demonstrated amplification and propagation of pacemaker Ca^2+^ signals by cADPR (58). A connection of cADPR signaling to Ca^2+^ entry was also observed: microinjection of cADPR in the absence of extracellular Ca^2+^ or in the presence of Ca^2+^ channel blockers resulted in much reduced Ca^2+^ signals (59). Finally, using a specific cADPR antagonist it was shown that downstream activation parameters of primary human T cells, such as activation antigen expression or proliferation, were concentration-dependently inhibited (14) suggesting a pivotal role of cADPR in T cell biology.

A detailed structure-activity analysis of cADPR in T cells has been conducted over the past couple of years. The main results from these studies were recently reviewed (60) and are (i) critical dependence of agonist vs. antagonist properties on the substituent at the C-atom 6 of the purine base, (ii) maintenance of biological activity, albeit at a lower level, when both southern and northern ribose were replaced by carbocyclic moieties or simplified ether/alkane bridges, and (iii) the possibility of radical simplification of the purine structure, e.g., the 1,2,3-triazole-4-amide mimic of adenine within cADPR retains biological activity.

## ADPR

A relatively new addition to the realm of adenine based Ca^2+^ mobilizing second messengers in T-lymphocytes is ADPR. Presence of ADPR in eukaryotic cells has been known for quite a while (61), but since ADPR is rather dangerous for the cell – its reactive ribose can non-enzymatically form Schiff-bases with amino groups of cellular proteins (62, 63) – it was mostly considered a toxic cellular waste product. This casually explained the presence of efficient mechanisms for the degradation of ADPR in form of cytosolic (64, 65) and mitochondrial ADPR pyrophosphatases (66, 67). These enzymes hydrolyze the pyrophosphate bridge of ADPR yielding AMP and ribose-5′-phosphate that are fed back into metabolism.

Two discoveries suggested that there might be more to ADPR: in 2001 it was reported that TRPM2 (formerly termed TRPC7 or LTRPC2), a Ca^2+^-permeable cation channel of the melastatin subfamily of TRP channels, can be activated by binding of ADPR to a cytoplasmic domain homologous to the mitochondrial ADPR pyrophosphatase NUDT9 (68, 69). This channel shows expression in a variety of tissues with highest levels being found in brain and cells of the immune system. A year later Bastide et al. showed that ADPR is also able to activate type I RyR isolated from rat skeletal muscle in the presence of micromolar concentrations of Ca^2+^ (70). Since there has been little news on the action of ADPR on RyR, we will focus on the role of ADPR for TRPM2 activation in T-lymphocytes.

Most of the work on ADPR and TRPM2 in T cells so far has been done in Jurkat cells that express TRPM2 on transcript and protein level and respond with a typical TRPM2 current to ADPR infusion (11, 45, 69). Microinjection of ADPR (11) and uncaging of photoactivatable ADPR (71) in these cells results in Ca^2+^ entry-dependent Ca^2+^ signals. By HPLC analysis the cellular ADPR concentration of roughly 40 μmol/L in resting Jurkat cells (72) was shown to nearly double after stimulation with high concentrations of concanavalin A (11).

There are different conceivable ways how this ADPR might be generated. CD38 expressed in Jurkat as well as primary T cells (73) can metabolize β-NAD^+^ and cADPR to yield ADPR (74). The topological paradox initially described for cADPR also holds true for ADPR (1). This paradox might be resolved by specific uptake mechanisms for ADPR as have been reported for erythrocytes (75, 76). Another possibility is the presence of CD38 in a type III orientation (2). While the contribution of CD38 to the increase in ADPR after stimulation is still unclear, the basal ADPR seems to be independent of CD38 as the murine T-lymphoma line BW5147 that lacks transcripts for CD38 (77) has even higher basal ADPR levels [73 μmol/L (72)] than Jurkat cells.

Another way that has been discussed for the production of ADPR is the consecutive action of poly-ADPR polymerase (PARP) and poly-ADPR glycohydrolase (PARG) (78). While PARP activity and poly-ADPR levels are quite low in non-stimulated cells, there is a constant turn-over due to the low *K_M_* of PARG [reviewed in (79)] that might contribute to the basal ADPR detected in Jurkat cells. Under DNA damaging conditions like strong oxidative stress the activity of PARP increases to such levels that a large part of cellular β-NAD^+^ can be metabolized as has been shown for DT-40 cells (80). Data for a range of cells suggest activation of TRPM2 by oxidative stress results in cell death by apoptosis (78, 81), most likely due to mitochondrial calcium overload and downstream activation of caspases [reviewed in (82)]. While murine CD4^+^ T cells also die after exposure to hydrogen peroxide, this apparently does not involve TRPM2 (83).

Interestingly there have been reports that in T cells PARP-1 activation can occur after TCR stimulation in a way independent of oxidative stress or DNA damage resulting in poly-ADP ribosylation of NFAT [(84, 85); reviewed in (86)]. It might be speculated that this increased pADPR turn-over will result in increased cellular ADPR and TRPM2 activation hinting to a possible role for ADPR/TRPM2 in TCR signaling. In accordance with this, naïve CD4^+^ T cells from the wild-type mice upregulated TRPM2 after stimulation with α-CD3/α-CD28-beads and CD4^+^ T-lymphocytes from TRPM2^−/−^ mice showed not only reduced proliferation, but also reduced production of pro-inflammatory cytokines upon activation (83).

Most work on the role of TRPM2 in the immune response has been done using the TRPM2^−/−^ mouse (87). In a model for ulcerative colitis the inflammation was suppressed, but this was shown to be due to a reduced production of the chemokine CXCL2 in monocytes whereas the infiltration of T cells in the colon was not affected by the knock-out of TRPM2 (87). Recent work has shown that TRPM2 knock-out does not affect airway inflammation either induced by oxidative stressors (88) or as a result of exposure to ovalbumin in a mouse model for acute asthma (89). On the other hand TRPM2^−/−^ noticeably reduced inflammation and spinal cord lesions in EAE induced by a peptide from myelin oligodendrocyte glycoprotein (83). Since the effect of TRPM2^−/−^ on EAE might also involve reduced neuronal cell death or microglia activation, it will be interesting to see whether proliferation and effector functions of T cells from such animals are affected.

Taken together, adenine derived Ca^2+^ mobilizing second messengers play essential roles in T cell Ca^2+^ signaling, both during activation/re-activation or apoptosis. While NAADP is important as rapid Ca^2+^ trigger, particularly in effector T cells, cADPR apparently holds a central role in maintenance of long-lasting Ca^2+^ signaling. Interestingly, also apoptosis induction via TRPM2 involves an adenine derived second messenger, the dinucleotide ADPR. The documented involvement of these adenine derived Ca^2+^ mobilizing second messengers in central aspects of immune regulation make the pathways described in this review suitable targets for therapeutic intervention. In fact, we have recently shown that the NAADP antagonist BZ194 ameliorated the clinical course of transfer EAE, an animal model of multiple sclerosis (40).

## Conflict of Interest Statement

The authors declare that the research was conducted in the absence of any commercial or financial relationships that could be construed as a potential conflict of interest.

## Abbreviations

ADPR, adenosine diphosphoribose; cADPR, cyclic ADP-ribose; [Ca^2+^]_i_, free cytosolic Ca^2+^ concentration; [Ca^2+^]_lu_, free endoplasmic reticular luminal Ca^2+^ concentration; CICR, Ca^2+^-induced Ca^2+^ release; EAE, experimental autoimmune encephalomyelitis; IP_3_, D-*myo* inositol 1,4,5-trisphosphate; IP_3_R, D-*myo* inositol 1,4,5-trisphosphate receptor(s); NAADP, nicotinic acid adenine dinucleotide phosphate; NAD(P), nicotinamide adenine dinucleotide (phosphate); pADPr, poly-ADP-ribose; PARG, poly-ADP-ribose glycohydrolase; PARP, poly-ADP-ribose polymerase; RyR, ryanodine receptor(s); Stim1, stromal interaction molecule-1; TPC, two-pore channel(s); TRPM2, transient receptor potential channel, subtype melastatin 2.
